# Advances in Medical Wearable Biosensors: Design, Fabrication and Materials Strategies in Healthcare Monitoring

**DOI:** 10.3390/molecules27010165

**Published:** 2021-12-28

**Authors:** Sangeeth Pillai, Akshaya Upadhyay, Darren Sayson, Bich Hong Nguyen, Simon D. Tran

**Affiliations:** 1 McGill Craniofacial Tissue Engineering and Stem Cells Laboratory, Faculty of Dentistry, McGill University, 3640 University Street, Montreal, QC H3A 0C7, Canada; sangeeth.pillai@mail.mcgill.ca (S.P.); akshaya.upadhyay@mail.mcgill.ca (A.U.); darrensayson@gmail.com (D.S.); 2 Department of Pediatrics, CHU Sainte Justine Hospital, Montreal, QC H3T 1C5, Canada; bich.hong.nguyen@umontreal.ca

**Keywords:** biosensors, wearable technology, biosensing materials, medical monitoring

## Abstract

In the past decade, wearable biosensors have radically changed our outlook on contemporary medical healthcare monitoring systems. These smart, multiplexed devices allow us to quantify dynamic biological signals in real time through highly sensitive, miniaturized sensing platforms, thereby decentralizing the concept of regular clinical check-ups and diagnosis towards more versatile, remote, and personalized healthcare monitoring. This paradigm shift in healthcare delivery can be attributed to the development of nanomaterials and improvements made to non-invasive biosignal detection systems alongside integrated approaches for multifaceted data acquisition and interpretation. The discovery of new biomarkers and the use of bioaffinity recognition elements like aptamers and peptide arrays combined with the use of newly developed, flexible, and conductive materials that interact with skin surfaces has led to the widespread application of biosensors in the biomedical field. This review focuses on the recent advances made in wearable technology for remote healthcare monitoring. It classifies their development and application in terms of electrochemical, mechanical, and optical modes of transduction and type of material used and discusses the shortcomings accompanying their large-scale fabrication and commercialization. A brief note on the most widely used materials and their improvements in wearable sensor development is outlined along with instructions for the future of medical wearables.

## 1. Introduction

Biosensors are devices that utilize biomolecular cues from analytes to process and produce quantifiable signals. The concept of “biosensing” surfaced around the early 20th century with the simple concept of acid concentration in liquids showing proportionality to the electrical potential across a membrane [1]. However, it was not until 1956 that a true biosensor device was developed by Leland C. Clark, Jr. for oxygen detection [2]. Thus began the evolution of an array of biosensor platforms ranging from fiber-optic-based detection of CO_2_, oxygen [3], and glucose [4], to the use of surface plasmon resonance (SPR) for gas detection [5], with the breakthrough of the first handheld blood biosensor (i-STAT) developed in 1992 [3]. Today, 20 years later, biosensors have made their way into our everyday lives in the form of wearable technology. Ranging from a simple fitness band that counts our daily steps, to highly multiplexed devices that detect non-abundant biochemical markers in body fluids, wearable biosensors have revolutionized our outlook on healthcare monitoring. This means, nowadays, it is possible for us to sit home and get real-time, dynamic information on our physiological functioning, just by a click, a tap, or a scan. These devices can capture different types of biosignals like changes in temperature, pulse, pH, motion, and biochemical composition of body fluids rapidly. In addition, many biosensors are currently developed as rapid, point-of-care devices that can be used in large-scale population screening to detect several viruses, including the most recent SARS-CoV-2 [6]. However, a sneak peak of the commercial market of wearable biosensors revealed a battle among the tech giants. A recent post on CBINSIGHTS highlighted that telehealth, wearables, and virtual reality will ace the battle of technologies and industries that shape a post-pandemic world [7]. An economical, easy-to-fabricate, high-throughput multimodal device with superior biocompatibility would naturally see a substantial growth in the industry, if made readily available, and can thereby make an appreciable impact on remote healthcare monitoring. For routine clinical applications, these materials should be comfortable and compatible with human skin surfaces and living tissues to constantly garner biosignals and generate computable results. In addition to ideal biointerfaces, wearable biosensors demand the use of materials that have superior sensitivity for correct recognition of analytes and high selectivity to identify specific types of environmental stimuli at a given time [8]. From the era of stiff electrochemical devices to the development of soft, flexible, and printable functional materials that adhere to jagged skin surfaces, there has been a paradigm shift in the way these modern materials are fabricated and utilized for biosensing applications [9]. In this narrative review, we will first describe an ideal biosensor in terms of the key components and sensing mechanisms while defining biointerfaces. We will then discuss the different types of wearable biosensors based on their sensing platforms, with a focus on the electro-chemical, mechanical, and optical mode of biosensing. We will mainly focus on the advances in biosensing materials and fabrication strategies used for developing these devices, with a brief note on the current challenges and future research focus in this area.

## 2. Design, Ideal Requirements, and Types of Wearable Biosensors

**Figure 1:**
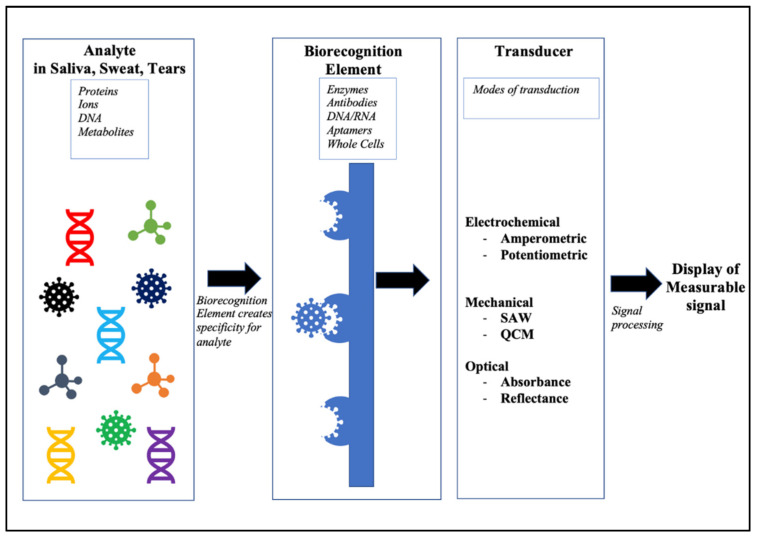
Schematic illustration of the basic parts of a biosensor and the working mechanisms. The analyte is usually the biomolecule that is recognized by the highly specific biorecognition element, and this reaction takes place on different transduction platforms, generating signals that are detected by transducers and converted into displayable data.

A biosensor is broadly made up of three main components: the biorecognition element, the transducer, and the signal amplifier (Figure 1). The two main areas of focus in this review are the biorecognition element and the transducer.

The success of biosensing lies within the recognition element. Typically, antibodies, enzymes, whole cells, and polymers have been used as the biorecognition element, as they allow for high specificity [10]. However, there are challenges when using such sensitive biomolecules—for example, the stability of the proteins for use in wearable devices tend to degrade over time and therefore can provide unreliable data. To overcome this challenge, recent biosensors prefer the use of aptamers, which, in addition to providing that high level of specificity and sensitivity, are also less prone to degradation [11]. These elements are immobilized using several physical and chemical strategies on the sensor to capture the target analyte. Biosensing materials such as hydrogels, graphene, and nanoparticles have gained widespread attention recently for their use as biorecognition elements and transducers [12]. Although biosensors have the same general mechanism for biorecognition, it is in the mode of transduction that they differ. The transducer is responsible for taking the reaction generated by the binding of the analyte to the biorecognition element and converting it to a readout that is proportional to the concentration of the analyte. Using a similar set of parameters as outlined by Luka et al. (2015) [13], this review will evaluate the advances made in wearable biosensors on the three mainly applied modes of transduction: electrochemical, electro-mechanical, and optoelectrical/photo-sensing platforms.

### 2.1. Ideal Requirements for Use as Wearable Biosensors

(a) Comfort: An essential element in the creation of a wearable biosensor is its flexibility and stretchability [14]. The ability to withstand and sense the strain is an important aspect of a device that will be subjected to constant strain and wear. With wearable biosensors that are often worn directly on the skin, there is a significant need for the materials to be adaptive and flexible. Materials that are not biocompatible with skin not only lead to general discomfort for the user, but also to low accuracy, as the biosensor–skin contact is not maintained for long periods due to the mechanical mismatch. Modifications such as the development of skin-inspired and patterned mesh to adapt to the human body’s curvilinear surface allows for better-adapted sensors [15]. Materials such as hydrogels, textiles, and paper have been mainstreamed for the development of wearable biosensors, as they provide a flexible, stretchable, and breathable platform for potential applications as wearable biosensors [16,17,18,19].

(b) Monitoring several analytes/parameters: An ideal wearable biosensor also has the capacity to monitor several biomarkers while maintaining physical properties. Different biosensor platforms have varying efficiency in measuring the analytes and the type of sample used. Some bioassays still use fluorescence or a colorimetric mode of detection, which poses a challenge to detecting low-abundance analytes.

(c) Biocompatibility: It is essential to fabricate the wearable sensor in such a way that the sensing surface that meets the skin is biocompatible and bioinert, and it must not cause a leakage, release toxic chemical molecules, or degrade due to wear and tear, and must provide adequate comfort while functioning accurately [15].

(d) Other considerations: Although not strictly a material requirement, there are other important considerations for developing a wearable biosensor. Miniaturized design, portability, scalability, and cost remain important factors in the creation of a wearable for widespread use. Although several low-cost and scalable methods have been presented in the literature, for example, devices made by screen-printing as a means of fabricating multiplexed biosensors [20], there is still a knowledge gap between developing a novel biosensor and actually applying it to large population groups.

### 2.2. Types of Wearable Biosensors

**Figure 2:**
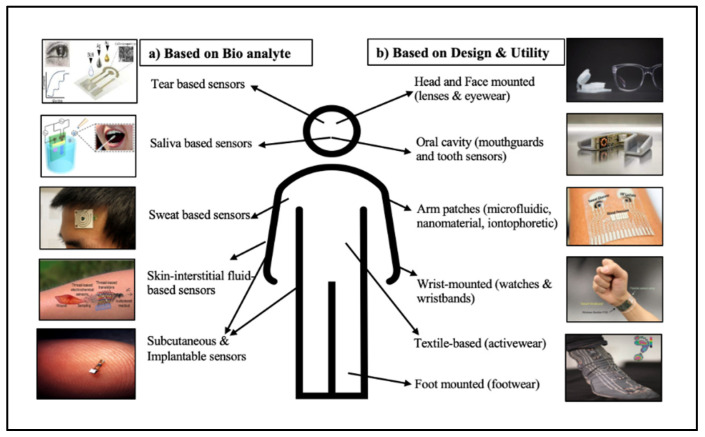
Different types of wearable devices categorized based on (**a**) bio-analyte and (**b**) design and utility. Images courtesy of [21,22,23,24,25], Reprinted with permission from [26] Copyright (2018), Elsevier [27,28], Reprinted with permission from [29]. Copyright (2019), American Chemical Society [30].

Within the literature, wearable biosensors are classified under several categories depending on their design and utility, material of choice, type of bioanalyte/biofluid used, or the transduction platform. Based on their design and utility, they can be divided into biosensors for the head and face or oral cavity, wrist- and arm-based wearables, textile based, and food mounted (Figure 2). Wearable biosensors can also be categorized based on the material they are made of, such as carbon based, inorganic biomaterials, or polymers, or categorized as flexible, biocompatible, or biodegradable sensors. Many authors tend to classify biosensors based on the biofluid/bioanalyte used, such as tear-, saliva-, sweat-, or interstitial fluid-detecting sensors, or based on invasiveness, such as subcutaneous and implantable sensors that utilize other physiological biomarkers to monitor health.

## 3. Electrochemical Biosensors

Biosensors that convert highly specific biologic information into electrical signals are an attractive tool in remote healthcare monitoring. Electrochemical biosensors were one of the earliest biosensor platforms to revolutionize wearable technology, as they use a fairly simple sensor setup and hence are cost efficient [31]. These benefits are associated with large-scale, low-cost electrical microcircuit production along with technology that allows easy readouts and data processing in these biosensors [31]. In addition, electrochemical biosensors have robust detection limits; even with limited analyte volume, they can produce accurate measurements due to their high specificity and binding affinity with the bioreceptors (e.g., enzyme–substrate or antigen–antibody interactions). These reactions, when converted into electrical signals, typically generate measurements as either (a) a current (amperometric), (b) a voltage or potential (potentiometric), or (c) a difference in conductivity of the electrolyte (conductometric) [32]. Other detection techniques described in the literature include impedimetric techniques, which combine both resistance and reactance [33] and the use of transistors to measure the current due to the potentiometric effect on the electrodes [31,34]. The most significant component of electrochemical biosensors is the electrode that holds the bioreceptor of interest. Therefore, it is crucial to employ a biocompatible and functional yet supportive material for their fabrication that allows for proper orientation, immobilization, and detection of analytes [35]. To fulfill these objectives, a variety of materials are tested across the labs to develop biosensors with the best performance. More insights on the available materials and their modifications are discussed under Section 7 (Material Considerations for Wearable Biosensors). In this section, we will mainly focus on the most recent developments in the field of electrochemical wearable biosensors and critically evaluate the possible future applications, and discuss the limitations that need to be overcome for their successful commercialization.

**Figure 3:**
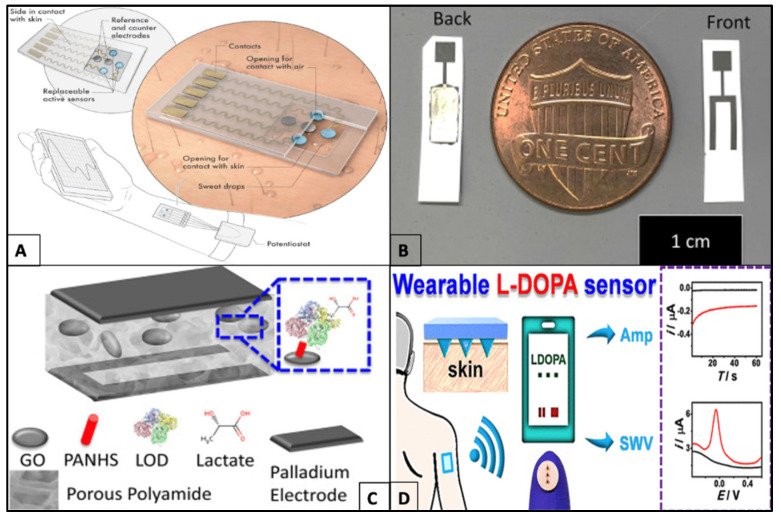
Examples of wearable electrochemical sensors. (**A**) Schematic illustration of the 2D MXene-based sweat sensor system showing the arrangement of electrodes, sweat uptake system, and sensors in contact with skin along with on-top openings for a sufficient supply of oxygen. Images reprinted with permission from [36], Copyrights (2019), John Wiley and Sons. (**B**) Image of a flexible graphene oxide (Flex-Go) biosensor in comparison to the size of a coin. (**C**) Schematic representation of the Flex-Go biosensor showing the electrodes (top—palladium and bottom—porous polyamide) with graphene oxide nanosheets embedded in between, along with 1-pyrenebutyric acid-*N*-hydroxysuccinimide ester (PANHS) and lactate oxidase (LOD) to detect lactate from sweat samples. Image reprinted with permission from [40], Copyrights (2021), Elsevier. (**D**) Schematic representation of a microneedle-based wearable patch for L-dopa detection from interstitial fluid (ISF) transmitting data to a wireless portable electrochemical analyzer sensed using square wave voltammetry (SWV) and amperometry. Reprinted with permission from [41]. Copyright (2019), American Chemical Society.

As described previously, depending on the type of analyte, different biosensing mechanisms and interfaces are used for their detection. Lei et al. (2019) described an MXene-based non-invasive, multifunctional, wearable sensor that uses a highly selective screening panel of biomarkers present in sweat [36]. They used a 2D MXene (Ti_3_C_2_T_x_) Prussian blue (PB) composite electrode design that has superior conductivity and electrochemical detection due to the solid–liquid–air tri-phase interface that combines with a hydrophobic substrate. The design of the biosensor is unique, as the tri-phase allows for better oxygen supply and thus increased stability of the sensor (Figure 3A). The sensors are designed to detect three main analytes: glucose, lactate, and pH levels, and the measured pH values are calibrated and plotted against the glucose and lactate concentrations to determine final results. They also conducted an in-vitro perspiration test on a human subject by connecting the wearable patch to an electrochemical analyzer and successfully measured the glucose, lactate, and pH changes during an intensive cycling session before and after meals. Results from this study show a promising wearable device with high performance owing to the 2D morphology and superior conductivity of the MXene/PB composite, stretchable and skin-integrating design of the patch with better oxygen supply, and the ability to analyze three different parameters simultaneously [36]. However, the fabrication and assembly of such a multiphase, multifunctional device might pose a challenge in terms of cost and commercial dissemination. In the same line, Yang et al. (2019) used a polyethylene terephthalate (PET)-based gold electrode (PGE) to develop an electrochemical sensor that also uses sweat as an analyte to detect glucose levels [37]. They used a UV-based chemical plating technique with a simple, cost-efficient, and easy-to-assemble sensor platform with a reliable amperometric output and high glucose sensitivity. This strategy overcomes the limitations of the previous generation of PET-based sensors that used a complex fabrication process and expensive techniques like photolithography and oxygen plasma etching, among others [38]. They also tested the efficiency of the PGE sensor with commercial glucose detection assays in testing sweat samples and observed comparable glucose concentrations [37]. Recently, an advanced, self-powered, sweat-based glucose-monitoring smartwatch was reported by Zhao et al. The smartwatch used flexible photovoltaic cells and batteries attached to a strap to generate a real-time, dynamic display of sweat glucose levels [39]. However, the complexity in designing a smartwatch with self-powering technology not only requires successful assembling of sensitive sensors and integrated display but also should align with current fashion trends while considering the cost. Lactate is another key bioanalyte used for fabricating electrochemical sensors due to its unprecedented role in monitoring several physiological and disease states.

Recently, Lin et al. (2020) developed a flexible graphene oxide (Flex-Go)-based biosensor for electrochemical lactate monitoring with sweat volumes as low as 1–5 Ul [40] (Figure 3B,C). Their research outcomes demonstrated that the dynamic range of a lactate biosensor is between 1–100 mM, which corelates to the physiological lactate levels in sweat and with comparable performance to their biosensor with a commercially available Lactate Plus meter [40]. However, although they established a benchmark for small-volume analyte detection, there are still several challenges that need solutions in terms of modulating the pH, dielectric strength, and conductivity of body electrolytes analyzed prior to focusing on the mass production and disposition of these wearables. Similar studies using lactate detection in sweat were performed by Wang et al. (2020) and Zhang et al. (2020), where both teams focused on developing flexible, non-invasive electrochemical sensors [42,43]. The Wang group developed a stretchable gold-fiber-based sensor integrated into a wearable textile that combined the high biocompatible, conductive properties of gold with superior lactate sensitivity in addition to high resistance against tensile strain [42]. Zhang et al., on the other hand, proposed the use of Ag nanowires (AgNWs) and molecularly imprinted polymers (MIPs) and showed the novelty by showing high detection limits of lactate of up to 0.22 μM in addition to reliable electrochemical response and stable electrode structure that can withstand repeated bending and twisting forces [43]. Despite their mechanical properties, compliance with the skin surface, and ease of mountability, they are still for proof of concept, but under appropriate technological guidance and marketing can pave the way to real-life applications.

Electrochemical sensors have recently seen increased growth as a drug-screening platform from body fluids, both for therapeutic and drug abuse. Barfidokht et al. (2019) developed the first “Lab-on-Glove” concept for rapid on-site detection of fentanyl with an electrochemical sensor integrated into the glove fingertips. The glove design utilizes the thumb to collect drug residues along with the index finger, which has printed carbon electrodes with an ionic liquid carbon nanotube composite film [44]. Voltametric outputs are generated on joining the thumb and index fingers, closing the electrical circuit, which results in anodic peaks that correspond directly to fentanyl oxidation levels. A similar strategy using carbon-based screen-printed electrodes (SPEs) has been used for other forensic applications, as they are cheap, disposable and have good compatibility with several electrochemical analyzers [45]. Several authors have used this technique to detect cocaine from body fluids and samples by targeting tertiary amine moiety of cocaine, which requires no incubation and has a rapid response [46,47,48]. Carbon-paper electrodes, including molecularly imprinted ones, have been widely used to develop electrochemical sensors for tetrahydrocannabinol (THC) sensing [49,50]. However, most of these sensors used for drug-abuse detection are still more focused on the “sensitivity” aspect of the assembly than the more futuristic wearable translation. Several electrochemical biosensors are gaining attention for use as therapeutic drug monitoring (TDM) to improve the pharmacokinetics of drugs with sensitive therapeutic ranges and poorly understood target doses. Takeda et al. (2020) developed a single-use, ceramic-based MIP using a carbon-paste electrode for monitoring drugs, including vancomycin, meropenem, theophylline, and phenobarbital. The relationship between drug concentration and response current was obtained using differential pulse voltammetry (DPV) [51]. Although the sensor was not fully non-invasive, owing to the requirement of blood as a sample to detect the drug of interest, their results suggest an efficient, cost effective, sensitive biosensor for remote (bedside) drug monitoring with minimal assistance. An example of a minimally invasive wearable electrochemical biosensor for monitoring levodopa was described by Goud et al. (2019). The multimodal microneedle patch works on two simultaneous parallel reactions: the enzymatic–amperometric and nonenzymatic–voltametric detection of l-dopa [41] (Figure 3D). Such a biosensor with superior analytical performance not only provides dynamic, accurate results due to its multidimensional data acquisition and processing, but also has potential to work with skin-like surfaces based on the results of tests on mouse-skin surfaces [41]. Such an orthogonal microneedle-sensing platform can be used for the detection of other biomarkers in interstitial fluids if the patch design is optimized to perform similarly on human skin. “The Lancet Digital Health” published in 2019 the first in-human evaluation of a microneedle-based biosensor for monitoring phenoxymethylpenicillin [52]. This proof-of-concept study tested real-time TDM in 10 healthy volunteers and concluded that a microneedle beta-lactam biosensor is highly effective and tolerated by healthy individuals. Evidence also shows that the pharmacokinetic profiles of phenoxymethylpenicillin were similar in microdialysis and microneedle methods. This study facilitates future research on microneedle ECF as a cornerstone in antibiotic drug monitoring. Although most of these studies show remarkable advances in terms of device form, flexibility, and well-integrated sensing modules, there are some noteworthy barriers that limit their application. We have yet to utilize the wide potential of new biomaterials used to develop these sensors due to lack of effective ways to fabricate and tune them to our needs. In addition, to utilize the potential of each material under various situations, we trail years of research in order to translate their use for health-monitoring purposes. More importantly, once these challenges are overcome, research focus should then be pointed towards making them safe to use, wear-resistant, and reusable, and possibly recyclable to meet the environmental regulations set in the future.

## 4. Electromechanical Biosensors

**Figure 4:**
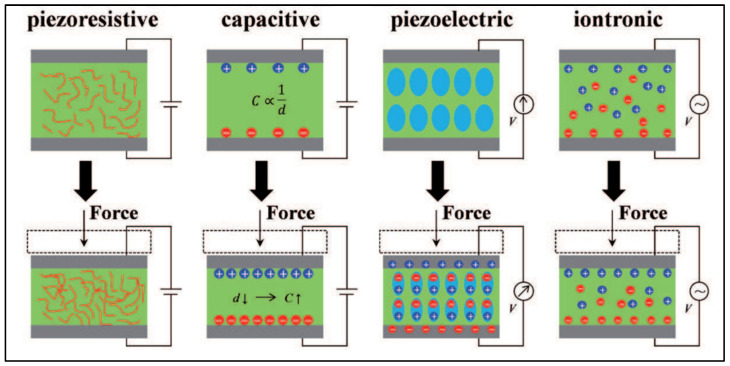
Schematic diagram showing different types of electromechanical transduction mechanisms. Image reproduced with permission from [8] Creative Commons Attribution 3.0 License.

An electromechanical biosensor works quite similarly to an electrochemical sensor in terms of the sensing principle; however, it varies in terms of how the electrical response is generated, which is chiefly due to a mechanical force or alternatively the strain recorded due to an electrical bias. A significant advantage of this type of transduction as opposed to electrochemical and optical sensors is the lack of dependence on the labeling of molecules. This can allow for the identification and quantification of a wide range of biomolecules, including unknown ones without the concern of lack of sensitivity or degree of analyte–bioreceptor interaction. Skin or epidermis is a soft and stretchable surface, and thus one of the most important mechanisms dictating the functioning of mechanical biosensors is their ability to sense the physical changes occurring on the skin surface at a macroscale, like arm, wrist, or leg movements or changes as small as a stretch or dampening of the epidermis like while breathing [53]. The electromechanical transduction mechanism is usually based on either (a) piezoelectric, (b) piezoresistive, (c) piezocapacitative, (d) iontronic, or I triboelectric nanogeneration (TENG) effects [8,54] (Figure 4).

**Figure 5:**
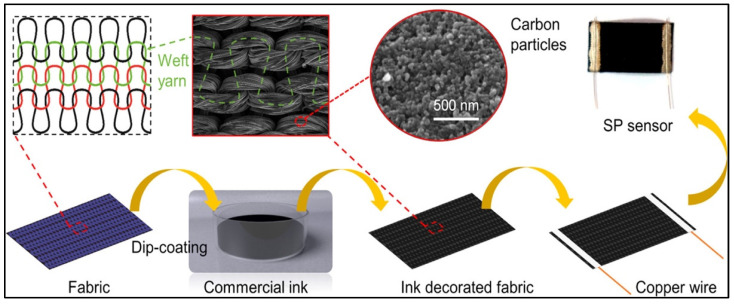
Schematic illustration of the fabrication process of an ink-decorated fabric strain sensor. First, the fabric is washed three times with deionized water, followed by dip coating in commercial ink, which is absorbed and dried in an oven at 60 °C for 1 h. Next, two copper wires are mounted onto the two ends of the fabric strip using silver paste to improve integration, which is dried again and ready for use with textiles. Reprinted with permission from [66]. Copyrights (2020), American Chemical Society.

Strain sensors function by quantifying mechanical deformation with corresponding changes to the electrical signals, which can be either piezoelectric (captures changes in surface potential due to polarization) or piezoresistive (captures changes in resistance caused by external forces) [53,55]. Capacitive sensors have superior sensitivity and react to changes in capacitance due to forces causing geometrical deformations; however, they are affected by surrounding noise, which may influence their performance [56]. Iontronic sensors utilize a supercapacitance platform that is about 1000 times larger than a metal oxide capacitor platform by forming an ion–electric interface between the electrodes and electrolytes, leading to ion accumulation on the electrodes and high capacitance per unit area [8]. Triboelectric transduction utilizes the simple principle of frictional charges that results from the interaction of two different materials. This was the principle used for the development of the TENG by Fan et al. (2012) [57]. When the friction is interrupted, the separation of the surfaces produces a difference in potential that is often used to develop sensors without use of external power [58]. Stretchable strain sensors require a high gauge factor to detect these minor movements, which occur on irregular skin surfaces and hence need to be designed with flexibility along with functionality. For example, Tang et al. (2020) designed a strain biosensor based on aligned nanowire with a high surface-to-volume ratio to monitor subtle human motion [59]. They were able to achieve a gauge factor of nearly 35.8 with the ability to detect a stimulus of a deformation of less than 200 µm in under 230 ms. The results were five times better when compared to a similar biosensor using microwire-based sensors [59]. In another study, Wang et al. (2020) created a stretchable ion-based sensor based on the surface strain redistributed elastic fiber (SSRE-fiber) that uses a wrinkle structure to improve surface area along with an island bridge design [60]. Although this principle does not use an electromechanical mode of sensing, the minimized strain on large amounts of stretching makes the SSRE platform an excellent choice for a textile-based wearable biosensor. Textile-based mechanical biosensors are an attractive tool in human motion detection and pave the way for personalized healthcare by functioning analogously to how we choose an outfit that conforms to our physical attributes. However, it is still a challenge, as such a technology would require the fabric to be highly conductive and equally resistant to strain. Usually, carbonization is the first line of choice to improve conductivity in textiles either by dip-coating, vacuum filtration, or the addition of metal nanoparticles [61,62,63,64,65]. Such an example of a wearable biosensor was developed using commercially available spandex by Yang et al. (2020), wherein they dipped the polyamide into carbonic pigment inks to fabricate a high-fidelity, conductive strain sensor [66] (Figure 5). Their work points to the potential application of these smart textiles by (i) mounting them to different joints (fingers, wrist, arm) to collect pulse rates, (ii) printing or sewing the strain sensors onto fabrics to capture functions like breathing and respiration, or (iii) utilizing them to generate protective devices for monitoring joint movements in sports medicine and posture-related diseases like Parkinson’s [66].

Tactile sensors, on the other hand, use the same transduction mechanisms but are fabricated to respond to external stimuli like pressure, touch, force, etc. [67]. These sensors are able to maintain their functional properties both in the native form and under deformation, which is mainly due to the bending (deflection) abilities and the high shape memory in response to multiplanar strains [67]. Our routine physiological response releases signals to the skin surface ranging from low frequency (~0.1–1 Hz) for respiration, facial expressions, and hand gestures to high-frequency acoustic signals (10–100 Hz) for speech and heart movements, utilizing the skin–air interface [68,69,70]. Jang et al. (2020) did a comprehensive update on pressure and tactile sensors for motion detection using field-effect transistors (FETs), which utilize the advantages of collecting and processing data in an array rather than individual sensors, allowing for high uniformity and spatial contrast to improve gesture recognition [71]. Such sensing mechanisms are essential to locate and collect large amounts of data from micro-expressions and split-second movements that occur on skin and superficial muscles and find key applications for the clinical diagnosis of several medical conditions. Ozioko et al. (2020) developed a piezoresistive wearable assistive tactile communication interface with vibrotactile feedback based on finger braille, a tactile communication platform used by deafblind people [72]. This work overcomes many challenges portrayed in the previous work on tactile braille-based communication where the sensors and actuators are isolated, making it difficult for users to interpret messages. The sensor showed maximum stability at 2.5 Hz cyclic loading, whereas the actuator can provide uniform vibrations with input signals of frequencies ranging from 10 Hz to 200 Hz, making the user feel and distinguish between messages [72]. On a different spectrum, pressure sensors are being widely used for applications in biomedical monitoring. Qi et al. (2020) reported the use of a core-sheath nanofiber yarn-based textile pressure sensor with superior sensitivity of 16.52 N^−1^, a wide sensing range (0.003–5 N), and rapid response time (~0.03 s) [73]. These properties will allow such pressure sensors to integrate into skin surfaces to accurately monitor human movements, from pulsations to tremors and high-intensity activities. Similar studies were reported elsewhere by Yi et al. (2021), wherein they used an all-3D-printed, flexible, and wearable hybrid bioelectronic tactile sensor fabricated using biocompatible nanocomposites. They show comparable results to the previously discussed studies with advantages of low detection limits, quick response rates, excellent biocompatibility, better compressibility, and a matching modulus of elasticity with human skin [74]. Developments in strain and tactile sensors have a multitude of applications, like monitoring general physical health, social interactions, assistance to specially abled populations, improving athletic performance and rehabilitation, and measuring sleep quality, among other applications. However, the human body releases an array of mechanical and/or acoustic signals for each action or physiological function, which sometimes occur simultaneously and are often superimposed, making it challenging to compute such high-frequency data signals. Although newer approaches like machine learning and advanced algorithms seem to overcome some of these concerns, the practical application and scope of wearable tactile sensors still requires a multimodal recognition approach to engineer precise gesture-control systems.

## 5. Optoelectronic Biosensors

**Figure 6:**
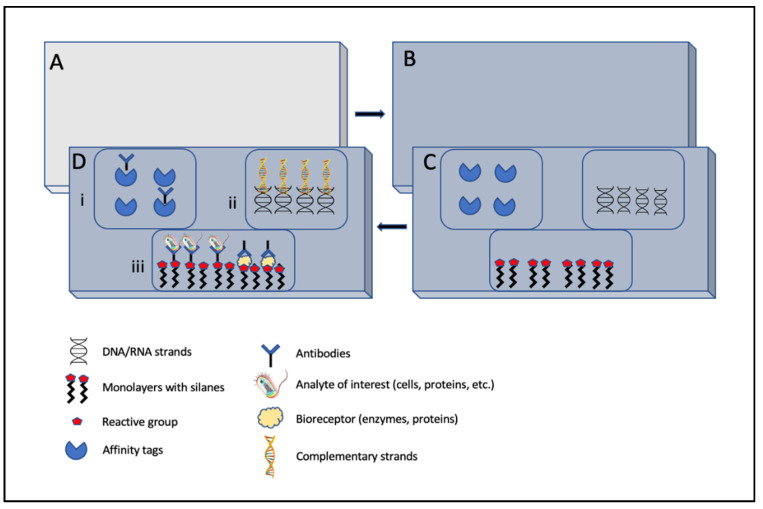
Schematic illustration of key biofunctionalization strategies for the fabrication of optical biosensors. (**A**) Cleaning of biosensor surface (grey surface). (**B**) Chemical activation of sensor surface (blue)—commonly used materials include silicon, its oxides, or nitrides, which use the silane chemistry to functionalize high-class optical transducers. (**C**,**D**) (**i**) Immobilization of bioreceptors on the treated surface, which can be affinity tags like protein A, a streptavidin biotinylated surface that attaches to selective antibodies; (**ii**) covalent immobilization (DNA-/RNA-based probes), which reacts with complementary strands; (**iii**) self-assembled monolayers with organosilanes to react with specific antibodies for covalent-based protein or analyte detection or hydrophilic monolayers with pegylated silanes or dextran-based molecules with covalently immobilized proteins for antibody detection. Image adapted with permission from [81], Copyrights (2011), John Wiley and Sons.

Optical biosensors detect or respond to analytes by undergoing optical changes like absorption, emission, reflection, refraction, or an interference [75]. These optical signals supersede the previously discussed physico-mechanical signals for transduction in terms of their high sensitivity, low noise, fewer surrounding disturbances, and long-term stability [76]. These advantages have led to several significant studies of applications in the field of diagnosis and drug discovery, health, and environmental monitoring [76,77]. In terms of optical transduction, sensor performance (sensitivity) is evaluated in terms of light-matter interaction, which corresponds to changes in optical signals and analyte changes. A large number of these biosensors exploit the evanescent wave phenomenon and can be routinely seen as a principle used in SPR-based biosensors (discussed in the next section), where they detect the changes in the refractive index on the sensor surface and appear as a shadow angled from the surface, depending on the mass of the material [78]. One of the main considerations while fabricating such optical biosensors is to focus on the biofunctionalization of the receptor on the sensor surface, which requires proper immobilization techniques for successful detection [79,80]. Usually, the first step in biofunctionalization is the cleaning of the sensor surface; next is the chemical activation of sensor surface; and third is the immobilization of the known specific biorecognition element, followed by the final detection stage, when target molecule attaches to the receptor (Figure 6). The immobilization may occur by simple physical adsorption, covalent bonding to the biomolecule, non-covalent or electrostatic interactions, or physical entrapment [81,82].

Several types of optical detection methods are described in the literature, but it is outside the scope of this review to detail them all. Thus, in this section, we will mainly discuss the most recent developments in SPR biosensors with potential remote healthcare applications, wearable fiber-based label-free and photoplethysmography (PPG)-based optical biosensors.

### 5.1. SPR-Based Optical Sensors

**Figure 7:**
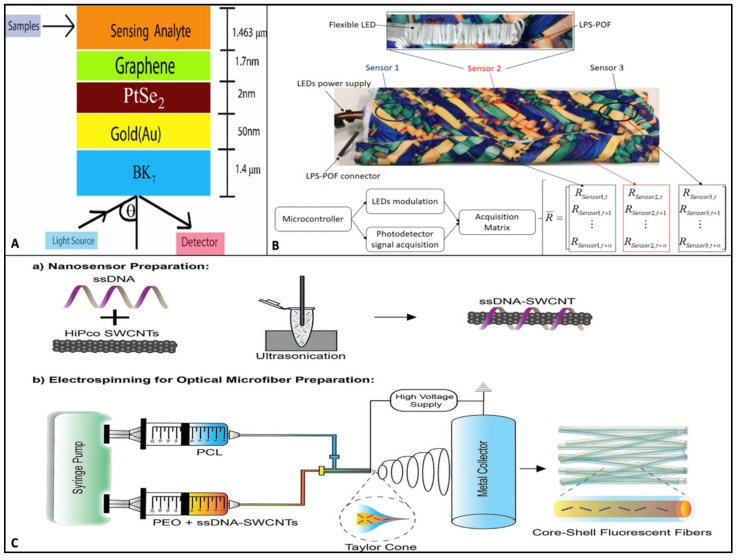
Optical biosensing technologies. (**A**) Schematic illustration of the multilayered SPR sensor made of BK_7_/Au/PtSe_2_/graphene layers for monitoring blood Hb and urine glucose levels. A monochromatic light source with a wavelength of 628.8 nm is applied to BK_7_ with a 60 deg to 89 deg angular range and the detector measures the sensor sensitivity based on the shifting of dip in reflectance intensity (%) based on variation in the refractive indices of blood and urine samples. Image reproduced with permission from [93], Copyrights (2021), Optical Society of America under the terms of the OSA Open Access Publishing Agreement. (**B**) Photographic representation of the multifunctional smart textile showing the LPS-POF sensor placement and schematic representation of the acquisition matrix due to sensor responses. Image reproduced with permission from [102], Copyrights (2020), Springer Nature. (**C**) Fabrication of optical microfibers: (**a**) Preparation of aqueously dispersed single-stranded DNA–single-walled carbon nanotubes (ssDNA-SWCNTs) by probe-tip sonication of SWCTs in the presence of ssDNA; (**b**) core-shell electrospinning setup for the fabrication of optical microfibrous textiles. Image reproduced with permission from [103], Copyrights (2021), John Wiley and Sons.

The past decade has seen tremendous growth in label-free SPR-based biosensor technology for biomarker detection and discovery due to its rapid, accurate, and versatile mode of action [83]. SPR is said to be the gold-standard transducer in the detection of several key biomarkers of heart disease, cancer, viral and bacterial antibodies, markers of hormonal regulation, renal function, and liver disease [84,85]. Although this technology has existed for decades, advancements in nanomaterials and fabrication strategies have seen newer sensors with enhanced sensitivity and a range of applications. Some SPR sensitivity improvements have been discussed recently [86,87,88,89]. Glucose monitoring has been quite successfully achieved using optical biosensors, as with the previously discussed electro-chemical and mechanical biosensors, and has been recently investigated by Zhang et al. (2021) [90]. However, this is achieved in a rather non-invasive and self-assisted manner with the optical transducers. Koushki et al. (2020) investigated the efficiency of optical glucose sensing in saliva using the SPR effect on gold nanoparticles (AuNPs) with a 10–13 nm diameter. They tested the effect of glucose and glucose oxidase (GOD) on the UV-vis spectrum of AuNps by either adding glucose solution to AuNps, followed by the addition of GOD or by the addition of glucose and GOD simultaneously, and measured the optical properties of both solutions. The glucose concentration in the tested solutions followed the sugar levels present in normal saliva [91]. The study concluded that the AuNP diameter is a key aspect in modulating SPR values, which suggests high glucose detection accuracy. These results can open potential applications of plasmonic nanometals in wearables like mouthguards and retainers for self-monitoring glucose levels in diabetic patients. On a similar note, with the aim of improving optical glucose detection, Yadav et al. (2020) did a comparative study by angle modulation for reflectance values in metamaterial (MM) SPR and SiO_2_-based SPR (Si-SPR) by fixing the wavelength and thickness constant [92]. They used urine samples to detect the sugar levels and found that the reflection dip angle varies between the urine of diabetic and non-diabetic patients with an angle of incidence at 50.7 and 60.1 deg between nondiabetic MM-SPR and Si-SPR. They further discovered the reflection angle to be different for diabetic patients with varying sugar concentrations. These data suggest progress towards the development of optical biosensors with specific sensor-surface modifications personalized to patients with different types of diabetic value ranges. More recently, Mostufa et al. (2021) developed a graphene-coated SPR biosensor for the detection of hemoglobin and glucose levels in blood and urine samples, respectively. The multilayered sensor was built with an initial layer of prism (BK_7_), followed by gold, a transition-metal dichalcogenide (TMDC) layer of PtSe_2_, and lastly, graphene (Figure 7A). Upon placing blood samples on the sensing layer, hemoglobin levels were detected by a 0.001 variation in a refractive index proportional to 6.1025 g/L hemoglobin in the sample. These values were used for analysis using COMSOL Multiphysics software and plotted using MATLAB software. Urine samples were analyzed by monitoring the SPR angle shift, and for a 0.001 refractive index change, the angular shift sensitivity was found to be 200 deg/RIU [93]. This multilayered–multianalyte detection SPR-based sensor can pursue a practical application in the bedside, remote monitoring blood hemoglobin levels and urine glucose simultaneously. A combination of 2D photonic crystal fiber (PCF) with SPR was developed by Mitu et al. (2021) for pregnancy testing by analyzing pregnant urine samples [94]. The approach was similar to the previous study using the finite element method (FEM) to numerically calculate the refractive indices, sensitivity, and resolution. Although SPR-based optical transducer modification and combinations have seen potential application in sensing applications, there is still a long way to go before making these advanced technical modifications into practical wearable sensors for remote healthcare monitoring.

### 5.2. Optical Fiber-Based Biosensors

There is a constant growth in fiber-optic technology across the industry and healthcare [95,96]. They have several merits, like compact size, low weight, immunity to electromagnetic radiation, robust and safe mode of action, superior sensitivity, and precision to function as wearables, among others [97,98]. These intrinsic characteristics of optical fibers have been well exploited by the developing wearable biosensing paradigm. Medical monitoring is the field that has benefitted the most due to this surge of interest and a great number of studies ae now focused on fiber-optic wearables.

Several commercial products have been developed, and many proof-of-concept studies have been conducted—for example, Li et al. (2020) devised an optical fiber-based, wrist-wearable, continuous and accurate blood-pressure monitor fabricated using a polydimethylsiloxane (PDMS) + Ag composite diaphragm and tested its functionality in 17 clinical subjects. The phase variation of the light reflected from this diaphragm surface was used to measure the blood pressure from the radial arterial pulse. The blood pressure values were obtained using the pulse transmit time (PTT), which was derived from the pulse waveform. On comparing the results with a routine sphygmomanometer, they found an error of 0.24 ± 2.39 mmHg and 0.12 ± 2.62 mmHg in the systolic and diastolic blood pressure, respectively, which is an acceptable variation in commercial BP monitors [99]. The proposed wrist band with optimized wearable design and display can work as a simple economic version compared to expensive smartwatch brands, and is a user-friendly device to continuously monitor BP and can replace current available commercial devices. Optical fibers using impact-detection systems usually use either a distributed or quasi-distributed approach to assess disturbances, and do so in the range of kilometers [100]. Distributed detection systems are either based on optical time-domain or optical frequency-domain reflectometry and are considered to have lower spatial resolution due to large distance range, in addition to expensive and nonportable components for sensor fabrication [101]. The quasi-distributed system, on the other hand, is based on the fiber Bragg gratings (FBGs) and provides a better spatial resolution due to shorter distance range, as low as centimeters between the FBGs, but still remains commercially inapplicable due to its bulky nature.

An alternative to this impact-based detection was proposed by Leal Junior et al. (2020) using transmission-reflection analysis (TRA) to develop a fully portable, wearable smart garment. Magnesium- and erbium-coated optical fibers in the sensor improve the challenges in spatial resolution reported previously due to low Rayleigh scattering when using single-mode fibers (SMFs) by increasing the Rayleigh scattering and, in turn, causing higher backscatter and better spatial resolution in TRA systems. Based on this approach, they developed a nanoparticle optical fiber (NPF)-integrated smart textile, which, along with TRA, allows for simultaneous detection of perturbation and fiber displacement due to external forces [104]. This is an excellent approach to developing a compact, portable system with low electromagnetic interference for continuous gait or natural posture monitoring in patients with movement or gait dysfunctionalities. The same group, Leal Junior et al. (2020), reported on the development of a multiparameter, quasi-distributed smart textile using highly stretchable polymer optic fiber (POF) fabricated using the light polymerization spinning (LPS) approach (Figure 7B). The textile was made of neoprene fabric comprised of LPS-POF along with three light-emitting diodes (LEDs) to sense variations in temperature, transverse force, and angular displacements [102]. The multiplexed sensor is capable of monitoring more than one parameter while maintaining its stretchable properties due to the LPS fabrication technique allowing for accurate movement detection without affecting the range of movements. More recently, Safaee et al. (2021) showed the fabrication of an optical core-shell microfibrous textile material incorporated with single-walled carbon nanotubes (SWCNTs) for real-time monitoring of reactive oxygen species (oxidative stress) in wounds by tracking the hydrogen peroxide concentration in wounds (Figure 7C). The SWCNTs have fluorescent properties that allow for continuous monitoring of oxidative species without succumbing to decay of signals [103]. Their application in textiles will allow for novel wound-dressing fabrics with the ability to monitor healing without degradation in optical properties. A similar stretchable, wearable, ultrathin sensor was developed by using a wavy optical microfiber by Zhu et al. (2021) using the bottom-up approach and was capable of successfully monitoring BP via wrist pulses [105]. Some other optical micro- and nanofiber-based wearable optical sensors used for monitoring respiration and bodily movements can be found in these papers [106,107].

**Figure 8:**
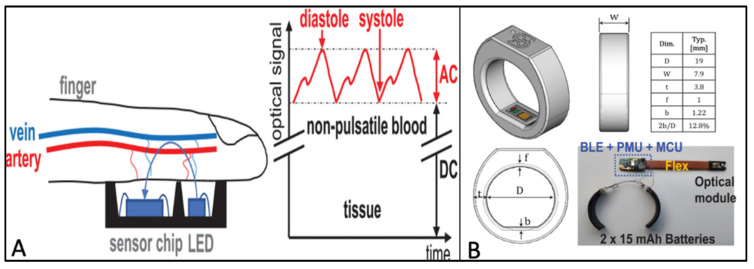
PPG-based optical wearables for heartbeat monitoring. (**A**) Schematic illustration of the sensor assembly for PPG measurement and the corresponding electrical components of the PPG signals. (**B)** Design of the ring at different views with a strapdown of the electrical module (right bottom); the top right shows the different parameters considered for superior ergonomics. Abbr.: low-energy Bluetooth (BLE), power-management unit (PMU), microcontroller (MCU), D (diameter), W (width), t (thickness), f (internal filleting radius), b (sensor position). Image reproduced with permission from [113], https://creativecommons.org/licenses/by/4.0/, accessed on 26 November 2021.

Fiber-free optical sensing and detection systems exist, including near-infrared spectroscopy to probe skin tissues to measure and analyze tissue oxygen saturation and blood flow [108,109]. A pulse oximeter is one such device, which is a non-invasive optical sensor working on the photoplethysmogram (PPG) technique used for blood volume (Hb) and blood oxygen (HbO_2_) changes [110,111]. In simple terms, the oximeters function by using two wavelengths of adequate path lengths to differentiate between absorption peaks of Hb and HbO_2_. Using this difference, measured as the ratio of light absorbed by each factor, the concentration or saturation of Hb and HbO_2_ is determined by the Beer–Lambert law [111]. A comprehensive literature review on the current developments in PPG-based label-free optical biosensors was discussed by Dhanabalan et al., 2020 [84]. A recent literature search also uncovered the potential of PPG in accurate heart activity monitoring, which is a searing topic among researchers for its use in remote evaluation of cardiac health. Recently Boukhayma et al. (2021) designed an earbud-embedded and a ring-embedded micro-powered millimeter-sized optical sensor for accurate heartbeat monitoring [112,113]. The monolithic optical sensor-based earbud uses a low-powered PPG chip fabricated in a 180 nm CMOS image sessor (CIS) with an area of 1.5 mm × 1.5 mm, which, due to the low dark current and noise, confers high sensitivity. The sensor module only utilizes a low 60 µA current at 122 Hz frequency and enables a high signal-to-noise ratio of PPG with an LED current of less than 10 uA. The ergonomic earbud design was tested clinically in human subjects and the accuracy was compared to a commercial electrocardiogram (ECG), which showed a 98.47% accuracy [112]. A similar module design strategy was employed to fabricate the ring-based sensor featuring the same quantum efficiency (QE) of 85%, and the detection rate was 97.87% for 72.21 h for heartbeat monitoring compared to the ECG [113] (Figure 8A,B). The miniaturized structure and low-power, long battery-based ergonomic design paves the way for such technology to be translated into commercially available medical sensors for remote medical care. However, there are still missing pieces to the puzzle in terms of design optimization and cost-effective fabrication of these optical sensors, alongside the practicality of device support while translating this technology to the commercial market. In this past section, we discussed the examples of most recent advances made in electrochemical, mechanical, and optical wearable devices. Table 1. provides an overview of these examples based on the type of biomolecule used, platform of wearable device, material and transduction platform, in addition to their intended applications and current challenges.

## 6. Material Considerations in Wearable Biosensors

### 6.1. Carbon-Based Sensors

#### 6.1.1. Graphene-Based Sensor Materials

Graphene has a honeycomb-like hexagonal carbon lattice arranged in sp2 hybridization, with sheets stacked in layers with van der Waals forces. Its peculiar structure gives it unique properties, like high electron mobility, good thermal conductivity, remarkable mechanical strength, and broad band light absorption. Graphene arranged in cylinders forms carbon nanotubes, and a in hollow sphere forms fullerenes. Limitations remain with its use given its low throughput yield and high cost, making its use restricted to research labs and academia [114]. Thus, there is a constant effort to build its hybrids, the most common being graphene oxide (GO) and reduced graphene oxide (RGO). Various sensing technologies developed using such hybrids include electrical sensors (resistive and FET (field-effect transistor)-based), electrochemical sensors, and optical sensors, including fluorescence-based sensors. A field-effect transistor (FET), as the name implies, functions by changes in the electrical current in the semiconductor channel in the presence of an ion on the sensor surface. The use of graphene in biosensors showed improved detection and sensitivity when compared to traditional bioassays [115]. Fu et al. (2017) achieved a detection level in picomolar concentration by the non-covalent functionalization of GO using HIV-related DNA hybridization [116]. Fluorescence-based sensors have also been explored for the detection of biomolecules. GO offers the dual advantage of being a fluorescent chromophore and a fluorescence quencher. A novel strategy was developed by Dong et al. (2010), where they utilized the interaction of GO with quantum dots (QD). Natively fluorescent QDs, functionalized with complementary DNA fragments, were quenched of their fluorescence upon their interaction with the GO surface. In the presence of the target DNA, this interaction was lost due to an increase in distance, thus sustaining the fluorescence [117].

**Figure 9:**
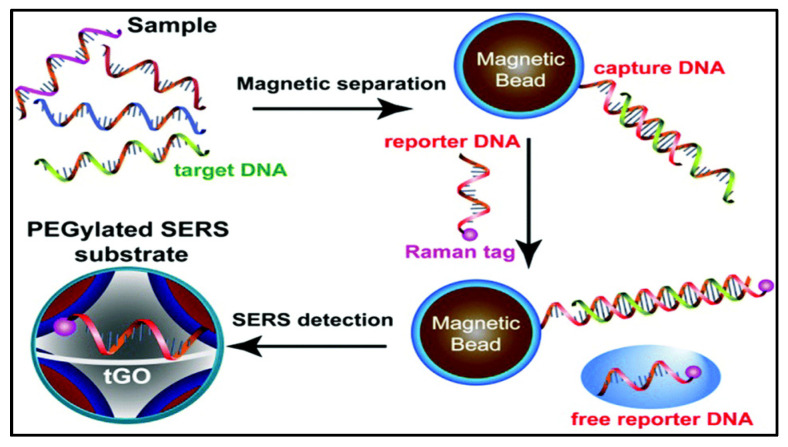
Mechanistic illustration of graphene-based substrates for SERS optical sensing. Image reprinted with permission from [119], under https://creativecommons.org/licenses/by-nc/3.0/, accessed on 26 November 2021.

Surface-enhanced Raman scattering sensing (SERS) is another optical sensing mechanism widely used in biosensors (Figure 9). The addition of graphene to SERS substrates provides better stability, sensitivity, and biocompatibility. Duan et al. (2015) developed a multilayered PEGylated (PEG: polyethylene glycol) nanosheet for multiplexed DNA detection, in particular for bacterial pathogens. Even with the wide range of research available on graphene-based sensors [118], there is still a long way ahead before they can be popularly integrated into day-to-day life, given the complexity, cost, and optimization required. Nonetheless, graphene-based materials offer high-quality sensing, which is worth the investment in terms of time, money, and resources.

#### 6.1.2. Carbon Dots

Carbon dots are an invention discovered serendipitously [120]. They are a zero-dimensional carbon-based material with a dimension of less than 10 nm. They have a tunable fluorescence emission, which makes it possible to adapt them to function in visible light and the non-infrared (NIR) region. This property is indispensable in biological environments, as this range is not damaging, as opposed to the UV spectrum. Their surface has oxygen-containing functional groups that allow for easy modification and molecule integration [121]. Their inert nature and stability in biological systems provide excellent biocompatibility and low toxicity. Generally, CDs can be classified into four categories: graphene quantum dots (GQDs), carbon quantum dots (CQDs), carbon nanodots (CNDs), and carbonized polymer dots (CPDs). CPDs are the newest inclusion, owing to the advent of organic monomer-based production of CDs using citric acid, urea, etc. CDs can be simultaneously used for imaging as well as drug-releasing pharmacologic functions [122]. The electrochemical-sensing mechanism utilizes the high electron transport capability of CDs for the sensitive detection of metabolites and ions [123]. Jiang et al. (2015) developed a dopamine biosensor, where dopamine reacted with the multiple carboxyl groups on the sensor surface, leading to a change in the electrical signal detected even at nanomolar concentrations [124]. Buk et al. (2019) immobilized glucose oxidase on a CQD–gold nanoparticle hybrid for highly sensitive glucose detection [125]. The optical sensing mechanism is also highly utilized in CDs, where they can be merged with nanoparticles like gold for increased signal production. CDs derived from phenylboronic acid utilized the interaction of glucose with surface boric acid to quench the fluorescence. It provided a sensitivity 250 times higher than previous boric acid nano-sensing detection systems [126]. Highly specific uric acid detection was utilized by Wang et al. (2016) with sulfur-nitrogen co-doped carbon dots [127].

In addition to direct detection, enzymatic activity can also be detected using substrate reactions rather than the target molecule itself. Such biosensors have been developed using both electrochemical [128] as well as optical-sensing mechanisms [129,130]. Other methodologies like antigen–antibody interaction [131] and nucleic acid [132] integration have also been explored using these highly versatile nano dots. HIV detection was achieved at a femtomolar level using this technological advancement [132].

#### 6.1.3. Carbon Nanotubes

**Figure 10:**
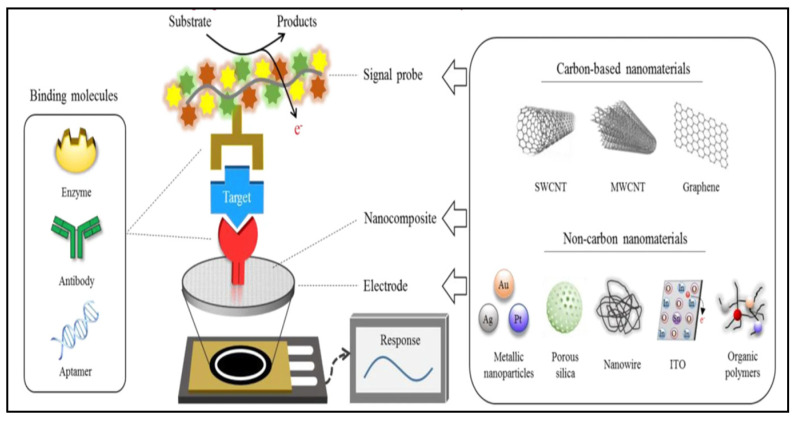
Mechanistic illustration of the principle of electrochemical biosensors based on carbon and non-carbon nanomaterials. Image reprinted with permission from [35], http://creativecommons.org/licenses/by/4.0/, accessed on 26 November 2021.

**Figure 11:**
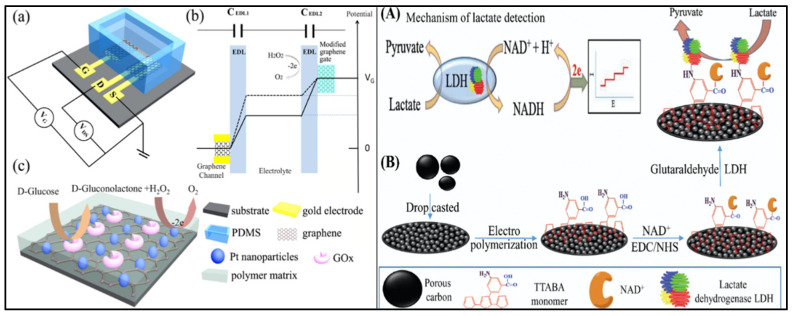
Mechanistic illustration of glucose (left) and lactate (right) detection. On the left, (**a**) schematic representation of a glucose sensor based on solution-gated graphene transistors (SGGT), (**b**) a drop in potential across the two EDLs (electric di-layers), (**c**) the GO-x catalyzed oxidation of glucose and oxidation of H_2_O_2_ on the electrode of the SGGT. Image reprinted with permission from [160], licensed under http://creativecommons.org/licenses/by-nc-nd/4.0/, accessed on 26 November 2021. On the right, (**A**) schematic illustration of the mechanism of lactate detection and (**B**) the fabrication of a lactate biosensor. Abbr. EDC/NHS (ethyl(dimethylaminopropyl) carbodiimide/*N*-Hydroxysuccinimide), TTABA monomer ((poly 3-(((2,2′:5′,2″-terthiophen)-3′-yl)-5-aminobenzoic acid). Image reprinted with permission from [161] Copyrights (2020), Elsevier.

Carbon nanotubes (CNTs) are an allotrope of carbon, which can be summarized as a cylindrical graphene. They have high mechanical strength, a large surface area, and excellent electrical and thermal conductivity [133]. However, there are strong π–π surface interactions, which makes them highly hydrophobic, limiting their functionality. Thus, activation is carried through various ways to increase their electrochemical properties, which in turn increase the sensitivity and often the specificity of the sensor [134]. They have been functionalized with various small molecules and enzymes for target-specific analyte detection, like nicotinamide adenine dinucleotide (NAD) [135], glucose oxidase [136], cholesterol oxidase [137], urease [138], and lactic-acid oxidase [139] (Figure 10 and Figure 11). CNTs are a vast field of research that cannot be covered completely in our review. More detailed discussions on CNTs, both single and multiwalled (MWCNTs), can be found in these papers [140,141].

### 6.2. Non-Carbon-Based Sensor Materials

#### Metal-Based Ceramics

(a) Silicon-based sensors: Silicon-based sensors include electrochemically active Si nanowires, optical porous silicone [142], photonic crystals, and luminescent quantum dots and wires. Their role in microfluidics for electrical, optical, and piezoelectric sensors has been studied and applied [143]. Electrical sensors are fabricated more commonly using Si nanowires and porous silicone. Porous silicone of 50–200 μm is used. Nanomolar levels of lipopolysaccharides (LPS) for bacterial presence along with high specificity was achieved by using an LPS-specific polymyxin B receptor probe. Song et al. (2007) developed a point-of-care cholesterol, bilirubin, and glutamate biosensor for liver diseases utilizing the increase in surface area of the porous silicone [144]. Meanwhile, the silicon nanowires provide a passive microfluidic-like structure due to capillary action, bringing down the detection limits to picomolar and femtomolar levels [145]. Optical sensing is also performed by the nanoporous silicone particles [146]. Photonic crystals (PhC) and silicon nanocrystals primarily function through optical pathways. FRET-based detection of *Staphylococcus aureus* was performed with a detection limit of 8 × 10^−14^ M using porous photonic crystals [147]. Silicon-based materials provide one of the most sensitive platforms for biosensing, but their manufacturing process and integration with other metal and non-metallic components remains highly demanding, thus limiting their largescale production and use.

(b) Zinc oxide (ZnO): Given the wide bandgap (3.37 eV) and high exciton-binding energy (60 meV) at room temperature, ZnO is one of the most ideal semiconductors for biosensors. Its high performance is owed to its excellent optical, piezoelectric, and electrochemical properties, along with the large surface area [148]. It is used in various shapes and sizes, ranging from zero dimensional QDs, one-dimensional wires and needles, and two-dimensional films, to three-dimensional porous materials and nanoclusters. There is a proof of concept for its use in neural [149], amino acid [150], and glucose-level [151] detection, as well as DNA hybridization [152].

(c) Aluminum oxide (Al_2_O_3_): Aluminum oxide-based materials are used in bioconductors due to their high dielectric constant, hardness, thermal stability, uniform pore size, and high pore density. Moreover, they are relatively less costly and the synthesis procedures are simple [153]. A CNT/Al_2_O_3_/chitosan hybrid was used to measure serotonin levels, which is an indicator of neural activity [154]. It has been used with GO [155], gold [156], polylysine [157], and titanium oxides [158] for various degradable as well as non-degradable biosensors.

### 6.3. Organic Materials

Natural biopolymer-based conductors mainly have two components: conductive fillers and an elastomeric polymer. Conductive fillers can be carbon based (graphene, CNT, CDs), metallic (metal oxides or noble metals), polymers and ionic liquids, or salts. The polymeric part can include synthetic polymers such as polydimethylsiloxane (PDMS), aka Ecoflex; polyurethane; polystyrene; or natural biopolymers—silk, gelatin, alginate, cellulose, or chitosan [159]. They have an advantage over other biosensing materials by being highly stretchable, mimicking biological tissues; are biocompatible; and are sustainable due to their biodegradability and natural abundance. The addition of conductive fillers imparts the required conductivity for imbibing electrical or optical properties. They provide wide applications from on-skin, textile wearable to implantable. They can be either bioinert or biodegradable, depending on the required conditions.

#### 6.3.1. Natural Polymers

(a) Polysaccharides: Cellulose is one of the most abundant polysaccharides present in nature, being present in early organisms like bacteria to more complex plants. It consists of branched polyglucan chains with multiple sites for hydrogen bonding, which imparts low solubility but high strength and flexibility at the same time. Cellulose-based biosensors have been widely studied for the detection of small molecules, nucleotides and DNA, proteins, glycoproteins, cancer and bacterial cells, and viruses. Chitin is the most abundant amino polysaccharide, where the amino groups make it possible to modify its properties in multiple ways. Chitosan is a deacetylated chitin derivative, with a more tunable structure, due to which it has acquired popularity over chitin in the biomedical research field. It has been used in conjugation with CNTs [162], graphene [163], and metals [164,165] for a wide range of molecular and cellular detection (Figure 11).

Another polysaccharide, alginate, is derived from brown seaweed with abundant use in biomedical applications. It can be modified into various forms, like fibers [166], sheets [167], and gels [168], as well as non-woven fabrics [169]. Ultra-high stretchability along with excellent fracture toughness is achieved by crosslinking with polymers like polyacrylamide (PAAm) and polyethylene glycol diacrylate (PEGDA) [170]. The dual detection of electrophysical and mechanical changes was achieved with the alginate PAAm hydrogel and polydimethylsiloxane (PDMS) film, paving the way for their use in biological situations involving coupled electromechanical reactions [171,172].

(b) Proteins: Protein-based natural polymers include silk and gelatin. Recently, silk was given FDA IDE approval owing to its biocompatibility [173]. It consists of two types of chains; one is fibrous, called silk fibroin (SF), and the other is stickier, called sericin. SF is usually utilized in biomedical applications, where it can be used as a substrate as well as a matrix for conductive filler materials. SF has hydrophilic amide groups, hydrophobic areas, and multiple functional groups, giving it the versatility to be conjugated with various filler materials. It has been integrated with gold nanoparticles [12], graphene(GO) [174], CNTs [175], and polystyrene sulphonate (PEDOT: PSS) [176] for various applications—for example, skin patches for electromyography (EMG) [177], cardiac mesh electrodes [178], and textile electrodes for physiological monitoring [176]. Biocompatible and conductive hydrogels crosslinked with gelatin have led to the development of biomimetic sensors that exhibit the advantages of natural polymers with enhanced physical and electrical properties. Gelatin meth acryloyl (GelMA) with DNA was used to conjugate with CNTs for increased strain tolerance [179]. Similarly, cross linking with polyvinyl alcohol (PVA) provided stretchability compared to natural skin, making it the ideal candidate for on-skin biosensing patches [180]. Though natural polymers have high biocompatibility, their use is not without drawbacks. Their complex structure and lot-to-lot variability limits their widescale manufacturing and use.

#### 6.3.2. Synthetic Polymers

Polymers can comprise a range of different functional moieties, like esters, urethane, phosphate, carbonate, amide, anhydride, and imide, such as poly(α-esters), polycaprolactone (PCL), polydioxanone, polyhydroxybutyrate, polyhydroxyvalerate, polyurethanes, and polyphosphazenes. Amongst polymers, esters like PCL and poly-glycolide (PGA) have been approved by the FDA due to their high biocompatibility [181]. Additionally, there is a class called intrinsically conducting polymers (ICP) that have an inherent electrical conductivity that can further be enhanced with organic and inorganic element addition. They include polypyrrol (PPy), polyaniline (PAni), and poly(3,4-ethylenedioxythiophene) (PEDOT) [181,182]. As mentioned at various places above, synthetic polymers have been used in conjugation with other conducting and optically active substrates to build a multitude of biosensing units [183]. Synthetic polymers can be more expensive than naturally available biodegradable polymers, but their advantage is a controlled synthesizing process. It ensures reproducible composition, electrochemical properties, and controlled degradation rates [183].

## 7. Conclusions and Future Perspectives

As presented in this review, the development of nanomaterials has resulted in novel ways to integrate biosensors into wearable devices while maintaining high selectivity of analytes and accurate biosensing. However, there are still challenges that remain in the development and eventual commercialization of such devices. The most significant hurdle that remains in fabricating these devices is employing materials and methods that are environmentally friendly, biocompatible, low-cost, and scalable. Although there has been significant development of biocompatible platforms and materials in sensors, there are still certain elements that limit the biocompatibility. Furthermore, some of these materials are prone to degradation over time and thus limit the life of the biosensor. With wearable biosensors, the nature of the functioning on the human body brings with it complicating factors such as temperature, moisture, and constant deformity. Each factor needs to be considered in the design to ensure proper functionality and accurate readouts. Apart from maintaining proper functionality, future wearable biosensors should incorporate additional features to create a user-friendly experience. In this review, we have provided a brief overview of the materials used in biosensing applications. Each material described has undergone extensive research with a plethora of literature, which is beyond the scope of our manuscript. Nonetheless, most important materials and their properties have been highlighted, which will serve as an essential tool for early researchers as well as other scientists. We can see how these materials can undergo various modifications and combinations to achieve the desired material properties. The goal is ultimately to build the most biomimetic tool possible that is biocompatible, highly sensitive, flexible, preferably compact, and adaptive to the dynamic biological and physical environment. Although there has been a great progress with respect to the materials, the cost and restricted access to technologies involved often limit their presence in research labs.

In the future, focus on better comfort, wireless communication, and sustainable power sources are a few of the challenges that need to be addressed to successfully integrate wearable devices. For example, although some wearables are battery powered, some take advantage of the energy generated by the human body that wears them to function. Further investigation on the use of solar cells and kinetic energy for powering wearable devices should be addressed. Another platform of recent interest is the rise of hydrogel-based biosensors that are deformable, biocompatible, and have self-healing properties, which addresses many of the challenges faced with stiffer 2D nanomaterials. Skin-printed electronic biosensors are an alternate, upcoming platform of biosensors that provides a viable solution to many of the issues discussed in our review, such as bulky sensor design, poor skin-surface interaction, the inability to accurately collect readouts, etc. Recently, a new wave of interest on wearable prosthesis has been largely welcomed by the medical community, alongside the tech industry. Intelligent robotic devices such as prosthetic limbs that can be gesture controlled or even respond to cerebral signals are being designed and normalized. With the rapid assimilation of AI into wearable devices, there should a focus on data collection and privacy policies that should be dealt with carefully to allow for safe and practical utilization. However, all these factors considered, more cost-efficient, easy-to-use, and accessible technologies for material and thereby device manufacture and development needs to be set in place to ensure their widescale progress and use in day-to-day biomedical applications. With further investigation into these technologies and their incorporation, the future of wearable biosensors is quite promising in biomedical applications, mainly in remote healthcare monitoring.

## Figures and Tables

**Table 1:** Selected examples of recently developed wearable biosensors.

Analyte, Parameter	Wearable Platform	Mode of Transduction	Materials	Application	Challenges	Ref.
Glucose, lactate, pH	Patch	Electrochemical	MXene (Ti_3_C_2_T_x_)—Prussian blue	Sweat monitoring	Fabrication and assembly due to its multiphase–multifunctional nature	[36]
Glucose	Mountable chip	Electrochemical	Polyethylene terephthalate (PET)	Sweat monitoring	Proof-of-concept study, needs optimization and validation to integrate into wearables	[37]
Glucose	Smartwatch	Electrochemical	PET	Sweat monitoring	Complex design and high cost of fabricating smartwatch components	[39]
Lactate	Mountable chip	Electrochemical	Graphene oxide	Sweat monitoring	Modulation of pH, dielectric strength, and conductivity of electrolyte and integration of wearables need validation	[40]
Lactate	Textile-based	Electrochemical	Gold fibers	Sweat monitoring	Textile shelf-life, fiber displacement, cleaning difficulties	[42]
Lactate	Skin-mountable chip	Electrochemical	Ag nanowires (AgNWs) and molecularly imprinted polymers (MIPs)	Sweat monitoring	Still a proof-of-concept study, needs validation studies for commercialization	[43]
Fentanyl	Gloves	Electrochemical	Printed carbon electrodes with an ionic liquid carbon nanotube composite film	Drug monitoring	Main focus on drug sensitivity, translation to wearable design at infancy	[44]
Vancomycin, meropenem, theophylline, phenobarbital	Bedside monitor	Electrochemical	Ceramic-MIP, carbon paste electrodes	Therapeutic drug monitoring	Partially invasive due to drug monitoring in blood	[51]
Levodopa	Microneedle patch	Electrochemical	Tyrosinase modified carbon-paste microneedle electrodes	Drug monitoring	Lack clinical validation and human skin biocompatibility tests	[41]
Beta-lactam	Microneedle patch	Electrochemical	Polycarbonate microneedles	Therapeutic drug monitoring	Proof-of-concept study, minimally invasive	[52]
Surface deformation	Mountable sensor	Electromechanical	Aligned nanowires	Motion detection	Proof-of-concept sensor, needs integration into wearable device	[59]
Sodium	Textile-based	Electrochemical–mechanical	Ion-based SSRE-fiber	Sweat monitoring	Lack of on-body trials, needs optimization for integration to textiles	[60]
Strain and conductivity	Textile-based	Electromechanical	Commercial spandex and carbon ink pigment-coated polyamides	Pulse rate, motion detection, and breathing	Textile/coated ink shelf life, cleaning, and multistep fabrication process	[66]
Vibro-tactile feedback	Finger–hand-based	Electromechanical	Velostat-polymer impregnated with carbon black	Tactile communication	Lacks longitudinal study to predict the interface success	[72]
Pressure sensations	Textile-based	Electromechanical	Ni-coated core-sheath nanofiber yarn with CNT-embedded polyurethane	Motion, pulse detection	Proof-of-concept design, needs optimization and validation for textile integration	[73]
Tactile sensations	Skin-mounted	Electromechanical	3D-printed nanocomposites	Motion detection	Proof-of-concept study; needs miniaturization to develop skin-compatible, compressible devices	[74]
Glucose, glucose oxidase	In-vitro model	Optical (SPR)	Au nanoparticles	Saliva monitoring	Proof-of-concept study, lacks integration into wearable device	[91]
Glucose	Bedside monitoring	Optical (SPR)	Metamaterial and SiO_2_-based SPR	Urine monitoring	High reliance on reflective dip angles, competing assays already in market	[92]
Hemoglobin and glucose	In-vitro model	Optical (SPR)	Prism (BK_7_), gold, PtSe_2_, and graphene	Blood and urine monitoring	Invasiveness, proof-of-concept studies, lacks integration into wearable devices	[93]
Reflectance due to pulse deformation	Wristband	Optical fiber	Polydimethylsiloxane (PDMS) + Ag composite diaphragm	Blood pressure monitoring	Optimization and display integration into wrist devices could be expensive	[99]
Perturbation and fiber displacement by external forces	Textile-based	Optical fiber	Magnesium- and erbium-coated nanoparticle optical fiber (NPF)	Motion and movement detection	Textile shelf-life, cleaning challenges	[104]
Temperature, transverse force, and angular displacements	Textile-based	Optical fiber	Polymer optic fiber (POF) using light polymerization spinning (LPS)	Motion and movement detection	Textile shelf-life, fiber disturbance during usage, and cleaning challenges	[102]
Hydrogen peroxide and ROS (reactive oxygen species)	Textile-based	Optical fiber	Optical core-shell microfibrous textile with SWCNTs	Wound monitoring	Novel design but patient comfort and wearable design considerations for site of wound	[103]
PPG-based tissue oxygen/blood saturation	Earbud	Optical fiber free	CMOS image sessor (CIS) with Bluetooth, power unit, and microcontroller	Heart rate monitoring	Commercialization and cost factors due to competing tech, and communication/power drawbacks needs to be addressed	[113]
PPG-based tissue oxygen/blood saturation	Ring	Optical fiber free	CMOS image sensor with Bluetooth, power unit, and microcontroller	Heart rate monitoring	Communication/power drawbacks and cost needs to be addressed for large-scale commercial applications	[108]
